# Phosphorylation-Dependent Pin1 Isomerization of ATR: Its Role in Regulating ATR’s Anti-apoptotic Function at Mitochondria, and the Implications in Cancer

**DOI:** 10.3389/fcell.2020.00281

**Published:** 2020-04-30

**Authors:** Yetunde Makinwa, Phillip R. Musich, Yue Zou

**Affiliations:** ^1^Department of Cancer Biology, University of Toledo College of Medicine, Toledo, OH, United States; ^2^Department of Biomedical Sciences, JH Quillen College of Medicine, East Tennessee State University, Johnson City, TN, United States

**Keywords:** cytoplasmic ATR, Pin1, antiapoptotic ATR, apoptosis, prolyl isomerization, cancer, cis and trans

## Abstract

Peptidyl-prolyl isomerization is an important post-translational modification of protein because proline is the only amino acid that can stably exist as *cis* and *trans*, while other amino acids are in the *trans* conformation in protein backbones. This makes prolyl isomerization a unique mechanism for cells to control many cellular processes. Isomerization is a rate-limiting process that requires a peptidyl-prolyl *cis*/*trans* isomerase (PPIase) to overcome the energy barrier between *cis* and *trans* isomeric forms. Pin1, a key PPIase in the cell, recognizes a phosphorylated Ser/Thr-Pro motif to catalyze peptidyl-prolyl isomerization in proteins. The significance of the phosphorylation-dependent Pin1 activity was recently highlighted for isomerization of ATR (*ataxia telangiectasia*- and Rad3-related). ATR, a PIKK protein kinase, plays a crucial role in DNA damage responses (DDR) by phosphorylating hundreds of proteins. ATR can form *cis* or *trans* isomers in the cytoplasm depending on Pin1 which isomerizes *cis*-ATR to *trans*-ATR. *Trans*-ATR functions primarily in the nucleus. The *cis*-ATR, containing an exposed BH3 domain, is anti-apoptotic at mitochondria by binding to tBid, preventing activation of pro-apoptotic Bax. Given the roles of apoptosis in many human diseases, particularly cancer, we propose that cytoplasmic *cis*-ATR enables cells to evade apoptosis, thus addicting cancer cells to *cis*-ATR formation for survival. But in normal DDR, a predominance of *trans*-ATR in the nucleus coordinates with a minimal level of cytoplasmic *cis*-ATR to promote DNA repair while preventing cell death; however, cells can die when DNA repair fails. Therefore, a delicate balance/equilibrium of the levels of *cis-* and *trans*-ATR is required to ensure the cellular homeostasis. In this review, we make a case that this anti-apoptotic role of *cis*-ATR supports oncogenesis, while Pin1 that drives the formation of *trans*-ATR suppresses tumor growth. We offer a potential, novel target that can be specifically targeted in cancer cells, without killing normal cells, to significantly reduce the adverse effects usually seen in cancer treatment. We also raise important issues regarding the roles of phosphorylation-dependent Pin1 isomerization of ATR in diseases and propose areas of future studies that would shed more understanding on this important cellular mechanism.

## Peptidyl-Prolyl Isomerization of Proteins and Pin1

**FIGURE 1:**
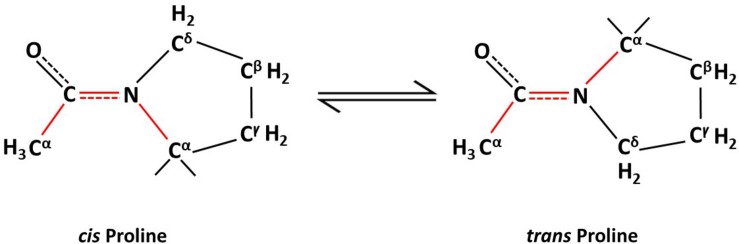
Non-enzymatic proline isomerization within proteins is a slow, rate-limiting process in the folding pathway.

Individual proteins may perform multiple functions and have evolved to evade unnecessary degradation. These differing functions and survival skills involve posttranslational modifications of proteins. Apart from protein function, post-translational modifications (PTMs) of proteins also can affect their sub-cellular location, stability and inter-molecular interactions with other proteins (Gothel and Marahiel, 1999; Lu and Zhou, 2007; Lu et al., 2007). Of the various types of PTMs such as phosphorylation, ubiquitination, acetylation, and so on, peptidyl isomerization of a protein is a unique type of PTM (Tanford, 1968). Peptidyl isomerization is the reversible transformation of a molecule between *cis* and *trans* isomeric forms, such that the peptide or protein can exist in two distinct geometric conformations, *cis* and *trans* (Figure 1). This modification causes no change in the molecular weight of the peptide or protein; hence, the inability to detect this change by mass spectrometry; however, isomerization, especially of a proline residue, alters the affected protein’s structure. The biological significance of prolyl isomerization, as compared to the other 19 non-proline amino acids, is that all non-proline amino acids are naturally stable in *trans* isomeric form whereas proline can be in either the *cis* or the *trans* isoform at the amide bond of proline with the preceding amino acid (Fischer and Schmid, 1990; Hinderaker and Raines, 2003; Song et al., 2006; Craveur et al., 2013; Figure 1). Thus, peptidyl isomerization of protein refers mostly to peptidylprolyl isomerization.

Most amino acid residues within a folded protein are thermodynamically more stable in the *trans* form (Stewart et al., 1990; Schmidpeter and Schmid, 2015). However, proline has the unique ability to exist as a *cis* or a *trans* residue in a protein’s structural backbone as the side chain of proline forms part of the backbone of protein (Fischer and Schmid, 1990; Hinderaker and Raines, 2003; Song et al., 2006; Craveur et al., 2013). This potential to switch between isomeric forms (Figure 1) *via* isomerization allows proline to act as a molecular switch that affects the protein’s structure and, hence, its physiological functions. The isomerization naturally occurs slowly and is rate limiting in the protein folding process. Hence, enzymes, such as peptidyl-prolyl *cis*/*trans* isomerases (PPIases) are required to overcome existing high-energy barriers between these protein isomers and to stabilize the transition between *cis/trans* isoforms. Protein isomerization is involved in many cellular processes such as apoptosis (Follis et al., 2015; Hilton et al., 2015), mitosis (Lu et al., 1996; Yaffe et al., 1997; Rippmann et al., 2000; Zhou et al., 2000; Yang et al., 2014), cell signaling (Brazin et al., 2002; Sarkar et al., 2007; Toko et al., 2013), ion channel gating (Antonelli et al., 2016), amyloidogenesis (Eakin et al., 2006), DNA damage repair (Steger et al., 2013), and neurodegeneration (Pastorino et al., 2006; Grison et al., 2011; Nakamura et al., 2012; Sorrentino et al., 2014).

**FIGURE 2:**
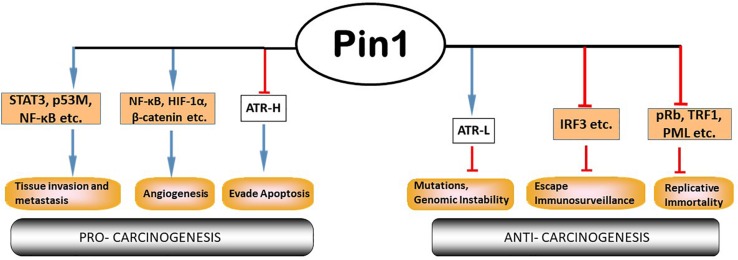
Pin1 participates extensively in multiple cellular processes involved in cancer. Pin1 has many cellular substrates that participate in the multi-step tumor development processes. Pin1’s roles can be contradictory: pro- or anti-tumor. Pin1 inhibits formation of *cis*-ATR and deprives the cell of *cis*-ATR’s anti-apoptotic role at the mitochondria, while promoting the formation of *trans*-ATR in the nucleus where it is important for repair of genotoxic stress to prevent mutations and maintain genome stability. Modified from Chen et al. (2018).

**FIGURE 3:**
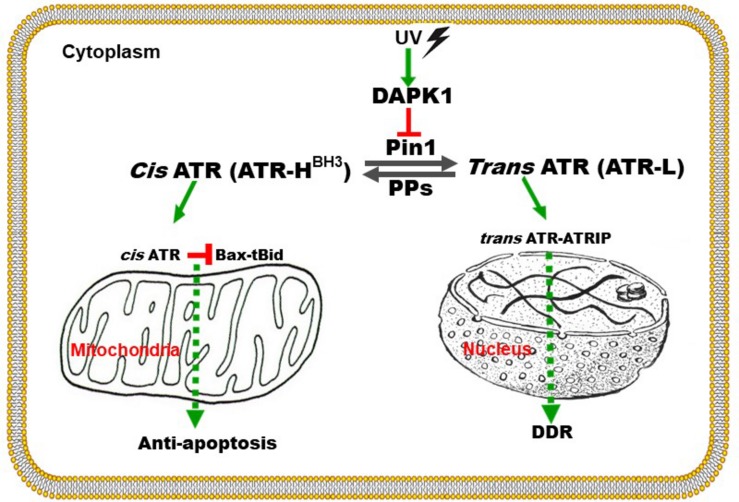
Graphical representation of the proposed mechanism by which ATR plays a direct anti-apoptotic function at the mitochondria. UV damage inactivates Pin1’s isomerization of ATR in the cytoplasm. *Cis*-ATR (ATR-H) then accumulates and binds to and sequesters t-Bid at the outer mitochondria membrane. Without tBid, Bax and Bak fail to polymerize, thus *cis*-ATR inhibits cytochrome c release and apoptosis. *Trans*-ATR (ATR-L) is the dominant isomer in the nucleus where it interacts with ATRIP, RPA and chromatin in the DNA damage repair (DDR) response. PPs (protein phosphatases) can dephosphorylate the Pin1 recognition motif and promote formation of *cis*-ATR (to be published elsewhere). Modified from Hilton et al. (2015).

Pin1 is a member in the parvulin family of peptidyl prolyl isomerases (PPIases); it can catalyze proline isomerization only at a phosphorylated Ser/Thr-Pro (pSer/pThr-Pro) motif (Lu et al., 1996, 2007; Lu and Zhou, 2007). Structurally, Pin1 consists of an N-terminal WW protein interaction domain which binds its substrate at the pSer/pThr-Pro motif, a central flexible linker and a C-terminal PPIase domain to catalyze proline isomerization (Lu et al., 1996). Pin1’s activity, stability, subcellular location and substrate binding can be regulated by its own PTMs, including Serine 71 phosphorylation by DAPK1 (inactivates Pin1; Lee et al., 2011; Hilton et al., 2015), ubiquitination (Eckerdt et al., 2005) oxidation (Chen et al., 2015), and sumoylation (Chen et al., 2013). Pin1 is involved in regulating multiple cellular processes including cell cycle transit and division (Rippmann et al., 2000), differentiation and senescence (Hsu et al., 2001; Toko et al., 2014) and apoptosis (Pinton et al., 2007; Follis et al., 2015; Hilton et al., 2015). To perform these cellular functions, Pin1 binds to many substrates within the cell (Figure 2). These substrates include proteins involved in cell cycle regulation (p53, cyclin E), transcriptional regulation (E2F, Notch1), DNA damage responses (DDR), and so forth (Lin et al., 2015; Chen et al., 2018). Pin1 expression and activity have been implicated in many diseases from neurodegenerative disorders such as Alzheimer disease and amyotrophic lateral sclerosis (Pastorino et al., 2006; Kesavapany et al., 2007; Nakamura et al., 2012, 2013), autoimmune diseases like systemic lupus erythematosus (Wei et al., 2016), to cancer (Ayala et al., 2003; Ryo et al., 2003; He et al., 2007; Yeh and Means, 2007; Finn and Lu, 2008; Nakamura et al., 2013; Lu and Hunter, 2014; Lin et al., 2015; Zhou and Lu, 2016; Chen et al., 2018; El Boustani et al., 2018; Nakatsu et al., 2019), etc. ATR (*ataxia telangiectasia*- and Rad3-related) protein, a master regulator and phosphatidylinositol 3-kinase (PI3K-like) protein kinase in DDR (Zou and Elledge, 2003; Cimprich and Cortez, 2008; Flynn and Zou, 2011), was recently reported to be a substrate of Pin1 for prolyl isomerization (Hilton et al., 2015). Given that ATR phosphorylates hundreds of proteins in response to DNA damage (Matsuoka et al., 2007), isomerization of ATR by Pin1 represents a new paradigm in understanding Pin1’s biological activities, which is the focus of this article (Figures 2, 3).

## Posttranslational Modifications of ATR for Its Respective Nuclear and Cytoplasmic Functions

ATR is a key DDR protein kinase that the cell employs to sense replicative stress and DNA damage. Following replication arrest and formation of single-stranded DNA (ssDNA), RPA coats the ssDNA and recruits ATR-ATRIP complex via ATRIP (ATR interacting protein). ATRIP is the nuclear partner of ATR and carries bound ATR along to the DNA damage site, where ATR is autophosphorylated at its T1989 residue (Cortez et al., 2001). This phosphorylated residue serves as a docking site for TopBP1 to significantly enhance the activation of ATR’s kinase activity (Burrows and Elledge, 2008; Mordes et al., 2008; Liu et al., 2011). ATR in turn activates several key downstream proteins, including p53 and other checkpoint kinases such as Chk1, leading to an S-phase cell cycle arrest for proper repair of the DNA damage or apoptosis in case of excessive damage (Cortez et al., 2001; Zou and Elledge, 2003; Sancar et al., 2004; Mordes and Cortez, 2008; Ciccia and Elledge, 2010; Nam and Cortez, 2011; Saldivar et al., 2017; Ma et al., 2019).

Recently, ATR was found to function in the cytoplasm and was described to play an important anti-apoptotic role directly at the mitochondria, independent of nuclear ATR and its kinase activity (Hilton et al., 2015). In contrast to nuclear ATR which always remains in *trans* form in complexing with ATRIP, cytoplasmic ATR in the absence of ATRIP exists in two forms, *cis* and *trans*, the existence of which depends on changing just one peptide bond orientation in ATR by prolyl isomerization. The balance between *cis* and *trans* cytoplasmic forms is regulated by Pin1, which catalyzes the conversion of *cis*-ATR to *trans-*ATR by recognizing the phosphorylated Serine 428-Proline 429 residues (pS428-P429) in the N-terminal region of ATR (Figure 3; Hilton et al., 2015). The activity of Pin1 favors the formation of *trans-*ATR, but inactivation of Pin1 by DAPK1 kinase upon DNA damage promotes *cis*-ATR accumulation at the mitochondria as *cis*-ATR appears to be naturally stable in cells. It is proposed that unlike its *trans* isoform, *cis*-ATR has an exposed BH3-like domain that allows it to bind to the pro-apoptotic tBid protein at the mitochondria. This binding prevents tBid from activating Bax-Bak polymerization which is necessary for the intrinsic apoptotic pathway. Hence, *cis*-ATR performs an anti-apoptotic role that allows the cells to survive long enough to repair its damaged DNA (Figure 3). However, this can be a double-edged sword that can play a role in carcinogenesis as discussed below. The newly discovered BH3 domain, a hallmark of apoptotic proteins, in ATR defines *cis*-ATR’s role in the apoptosis pathway (Figure 3).

## Phosphorylation-Dependent Isomerization of Atr by Pin1

Pin1 has a high degree of phosphate specificity (Zhou et al., 1999; Lu, 2000; Liou et al., 2011). Due to the numerous amounts of phosphorylated substrates that Pin recognizes in the cell, Pin1 can be a potential target in treatment of many diseases (Ryo et al., 2003; Kesavapany et al., 2007; Finn and Lu, 2008; Liou et al., 2011; Lu and Hunter, 2014; Lin et al., 2015; Wei et al., 2015, 2016; Campaner et al., 2017; Chen et al., 2018). Since Pin1’s activity on ATR requires the phosphorylation at Ser428 of ATR, this could serve as an important regulatory tool to influence the levels of the ATR isomer. Thus, phosphorylation at Ser428 may play a critical role in regulating ATR prolyl isomerization and, thus, ATR’s anti-apoptotic activity at mitochondria.

**FIGURE 4:**
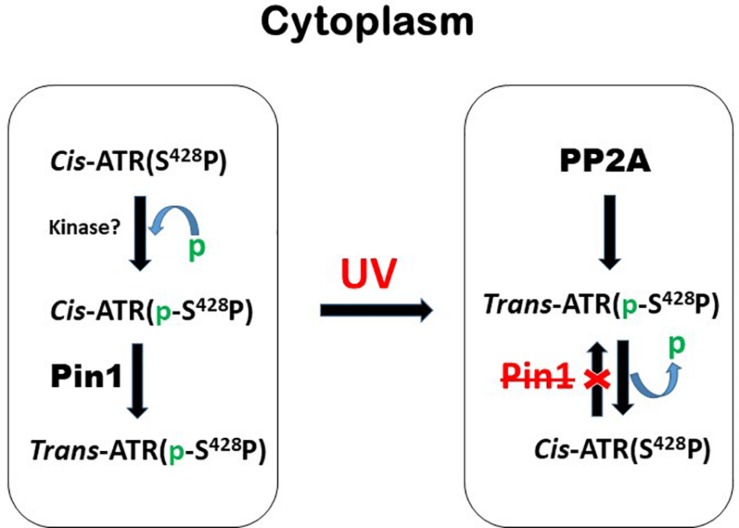
A brief summary of the mechanism by which the levels of cytoplasmic *cis*- and *trans*-ATR isoforms are mediated by phosphorylation and dephosphorylation before and after UV irradiation. The red X stands for inhibition or inactivation of Pin1.

Hilton et al. (2015) showed that when the serine 428 residue in human ATR is mutated to alanine (S428A), Pin1 is unable to recognize its motif to isomerize *cis*-ATR to *trans*-ATR; hence, cytoplasmic S428A ATR exists primarily as the anti-apoptotic *cis* isomer. In addition, when the proline 429 residue was mutated to alanine, the P429A ATR in the cytoplasm was in the *trans* form. This indicates that the type of ATR present in the cytoplasm can be regulated by targeting this phosphorylation-dependent Pin1-mediated isomerization of ATR (Figure 4). An accumulation of *cis*-ATR at mitochondria confers a survival signal that allows the cell to escape apoptosis even following DNA damage. The evasion of cell death may allow mutations that have occurred in these cells to be passed to daughter cells. Survival of an increasing number of cells with accumulating mutations over time can increase genomic instability and cause carcinogenesis. The alternative scenario where *trans*-ATR is dominant in the cytoplasm leads to an increase in free t-Bid since *trans* ATR is unable to bind and sequester t-Bid, allowing the programmed cell death that occurs when the cell is unable to repair DNA damage. In support of this mechanism proposed by Hilton et al. (2015), Lee et al. (2015) observed that a low expression of cytoplasmic pATR (S428; which implies higher levels of cytoplasmic *cis*-ATR) is associated with an advanced stage epithelial ovarian carcinoma (EOC) with poor disease prognosis and treatment outcomes. In contrast, no such correlations were found with nuclear pATR (S428) levels, implicating that cytoplasmic *cis*-ATR levels are uniquely important in the disease progression of EOC.

The level at Ser428 phosphorylation in ATR can be determined by two important classes of proteins: protein kinases and phosphatases. The former phosphorylates Ser428 while the latter dephosphorylates this residue. The balance between the two opposing activities is critical to controlling the *cis*/*trans* balance of ATR isomers and, thus, the health of the cells. Identification of the phosphatases which have activities at Ser428 is particularly important to cancer treatment as dephosphorylation of this residue leads to an increase of anti-apoptotic *cis*-ATR formation (Hilton et al., 2015) and poor prognosis for cancer treatment (Lee et al., 2015). Thus, the responsible phosphatase(s) would be a reasonable target for inhibition to improve cancer treatment. Indeed, we recently identified PP2A (Protein Phosphatase 2A) as the protein phosphatase that dephosphorylates Ser428 in the Pin1 recognition motif of cytoplasmic ATR. When PP2A dephosphorylates this Ser428 residue, Pin1 can no longer recognize its motif to isomerize cytoplasmic ATR from the *cis* to the *trans* isoform (Figure 4). This key regulation was found to increase the level of *cis*-ATR in the cytoplasm and its accumulation at the mitochondria to bind tBid for its anti-apoptotic role (Figure 3). In addition, cells in which PP2A was inhibited were found to be significantly more sensitive to DNA damage agents. In contrast, a kinase that phosphorylates cytoplasmic ATR at Ser428 in the Pin1 recognition motif will cause an opposite effect; in the cytoplasm, there would be a relative abundance of phosphorylated substrate for Pin1 to perform its phosphorylation-dependent isomerization of *cis*-ATR to the *trans* form. Since the *trans* form has no direct anti-apoptotic benefit following DNA damage, the cells with a predominance of cytoplasmic *trans*-ATR will succumb more quickly to apoptosis. It is worth noting that UV irradiation reduces the Ser428 phosphorylation level of ATR in the cytoplasm (Hilton et al., 2015) while at the same time increasing the phosphorylation level at the same S428 residue of ATR in the nucleus of cells. The former consistently leads to accumulation of *cis*-ATR at mitochondria. The latter’s effect remains unknown as the nuclear phosphorylation of ATR-Ser428 has no effect on ATR checkpoint activation of Chk1 after UV damage (Liu et al., 2011). In addition, while the mechanism of ATR isomerization is defined with the cells treated with UV, Hilton et al. (2015) also show that other types of DNA damage agents such as hydroxyurea and camptothecin can induce formation of *cis*-ATR in the cytoplasm though less efficiently. This suggests that the mechanism defined by Hilton et al. (2015) may represent a universal pathway of ATR isomerization in response to DNA damage. By simply regulating a PTM event in the cytoplasmic ATR protein, i.e., addition or removal of a phosphate group in the Pin1 motif of ATR, one would be able to control how cells respond to a DNA damaging event: survival or death as summarized in Figures 3, 4.

## *Cis*-Atr’s Anti-Apoptotic Function May Support an Oncogenic Process in Dividing Cells

Cancer is characterized with deregulated cell growth, where there is an imbalance in the inherent cell cycle regulation to check the rate and integrity of cell division and growth. *In addition, given that cis-ATR is antiapoptotic, we hypothesize that cis-ATR may perform an oncogenic role, while Pin1 might be tumor suppressive in terms of ATR’s anti-apoptotic activity at the mitochondria.* If *cis*-ATR is the dominant cytoplasmic form, it may block mitochondrial apoptosis and allow damaged cells to survive and mutate, even when DNA damage repair is insufficient and the abnormal cells are supposed to die via apoptosis. This evasion of apoptosis is an important hallmark of cancer cells that, over time, allows them to accumulate the mutations that define genome instability and, eventually, leads to carcinogenesis. However, if Pin1’s action is increased and *trans*-ATR is the dominant form of ATR in the cytoplasm, before mutations can be propagated, programmed death will occur in those cells that are too severely damaged for proper DNA repair. Thus, reduction of cytosolic *cis*-ATR discourages accumulation of cells with DNA damage that could be passed on to daughter cells and would promote carcinogenesis.

This hypothesis is interesting in and of itself, but is inconsistent with the existing literature which suggests other roles of Pin1 in cancer development (Figure 2). The current understanding stems primarily from observations that Pin1 is overexpressed/has increased activity in most cancers and cancer stem cells, with corresponding negative prognostic outcomes (Ayala et al., 2003; He et al., 2007; Tan et al., 2010; Girardini et al., 2011; Luo et al., 2014; Rustighi et al., 2014; Xu et al., 2016; Nakatsu et al., 2019). Also, Pin1 upregulates many oncogenes, while downregulating several tumor suppressor genes (Chen et al., 2018). Pin1 overexpression or its over activation can be inhibited by genetic approaches or chemically with juglone (Hennig et al., 1998), all-trans retinoic acid (ATRA; Toledo et al., 2011) or KPT-6566 (Campaner et al., 2017) and, when tested, Pin1 inhibitors were able to suppress cancers (Estey et al., 1997; Budd et al., 1998; Shen et al., 2004; Lu and Hunter, 2014; Wei et al., 2015; Zhou and Lu, 2016; Lian et al., 2018). However, there are many challenges to chemically inhibiting Pin1, especially with retinoids (e.g., ATRA), the most commonly used clinical inhibitor. These include low drug bioavailability, clinical relapse and retinoid resistance, etc. (Muindi et al., 1992; Decensi et al., 2009; Arrieta et al., 2010; Moore and Potter, 2013; Jain et al., 2014). In contrast, bioinformatic analyses of human tumors (Kaplan–Meier Plots) reported in the Human Protein Atlas (7,932 cases) found that low Pin1 RNA expression is largely associated with a lower survival profile for most types (12 types) of cancer patients while high expression correlates with a higher survival profile for three types of cancer (Table 1). For two other types of cancer the relationship of survival profile with Pin1 expression is non-determined. Interestingly, two types of male-only cancer, prostate and testis, are among the three types of minorities; these patients had a higher survival profile with low versus high Pin1 RNA expression. These results also are consistent with the 5-year survival probabilities (Table 1). However, of all the 17 cancer types analyzed, only in two types, renal and pancreatic, are Pin1 expression prognostic: high Pin1 expression is favorable for better prognosis as determined by Human Protein Atlas (Table 1). This appears to contradict a recent report on the prognostic value of Pin1 in cancer which analyzed the data from 20 published papers (2,474 patients) which concluded that Pin1 overexpression was significantly associated with advanced clinical stage of cancer, lymph node metastasis and poor prognosis, although no correlation with poor differentiation was found (Khoei et al., 2019). Interestingly, it is known that over 50% of cancers have mutations in p53, and Pin1 expression was found to promote mutant p53-induced oncogenesis (Girardini et al., 2011). Also, importantly, Pin1 isomerizes wild-type p53 in DDR and the wild type p53 functions are regulated by Pin1 (Wulf et al., 2002; Zacchi et al., 2002; Zheng et al., 2002). Thus, p53 status may affect the relationship between Pin1 expression and cancer as Pin1 appears to have different effects on cancer cells with mutant and wild-type p53 (Mantovani et al., 2015). It remains unknown if or how the p53 status would affect cancer prognosis in correlation with Pin1 expression levels, which is of great interest to determine. We propose that a wider role for Pin1 and its regulator partners in carcinogenesis needs to be considered and investigated further to provide better context (Han et al., 2017).

**TABLE 1:** Pin1 RNA expression in caner patients analyzed by Kaplan-Meier Plot (Human Protein Atlas).

Cancer type	Male/female (n/n)	Max post- diagnosis years	Pin1 expression						
			
			Survival probability		5-year survival (%)				
							Expression		Prognosis
					Low	High	Level		status
			Lower	Higher	expression	expression	cut-off	*P* score	(Prognosability)
Renal	591/286	16	Low	High	64%	82%	9.65	0.000078	Yes
Pancreatic	96/80	7	Low	High	7%	48%	8.72	0.00032	Yes
Glioma	99/54	7	Low	High	5% (^∗^)	12% (^∗^)	15.74	0.022	No
Thyroid	135/366	15	Low	High	91%	100%	9.19	0.031	No
Lung	596/398	20	Low	High	40%	47%	6.16	0.029	No
Stomach	229/125	10	Low	High	26%	50%	8.03	0.022	No
Breast	12/1063	23	Low	High	81%	82%	7.16	0.25	No
Cervical	0/291	17	Low	High	59%	74%	10.81	0.0061	No
Endometrial	0/541	19	Low	High	70%	80%	8.61	0.044	No
Ovarian	0/373	15	Low	High	27%	38%	13.22	0.0072	No
Urothelial	299/107	14	Low	High	33%	43%	7.49	0.012	No
Head and Neck	366/133	17	Low	High	39%	57%	8.75	0.0065	No
Melanoma	60/42	5	High	Low	37% (^∗^)	0 (^∗^)	15.17	0.27	No
Prostate	494/0	14	High	Low	100%	97%	11.77	0.094	No
Testis	134/0	20	High	Low	100%	97%	8.63	0.26	No
Liver	246/119	10	Non-determined		53%	46%	5.4	0.190	No
Colorectal	322/275	12	Non-determined		63%	60%	8.76	0.065	No
Total Cases	3679/4253			Low:High=3:12	(^∗^): 3-year Survival				

While it is logical to target Pin1 or the many processes that Pin1 regulates directly or indirectly via its substrates involved in carcinogenesis (see Figure 2), we propose that it would be significantly more effective to target the control of apoptosis, a common pathway always deregulated in carcinogenesis with uncontrolled proliferation. This is because apoptosis is the ultimate terminator and always has the final say in determining the fate, death or survival, of cells. This would tie in with the emerging idea of oncogene addiction, where the so-called “Achilles heel” of a cancer is used to deal a deathblow to that cancer (Weinstein, 2002; Weinstein and Joe, 2006, 2008). Oncogene addiction is one of the themes that has evolved in the study of tumor progression. There are innumerable causes of cancer, hence the difficulties in identifying suitable treatment targets for developing effective therapies. Research has shown that oncogenes and tumor suppressor genes are constantly undergoing mutations in the background of genetic instability that can drive tumor progression. Oncogene addiction attempts to simplify the essence of carcinogenesis to a single, most important oncogenic protein that a tumor depends on for its survival, while the counterpart normal protein has little or no negative effects on normal cell survival. If this oncogenic pathway is targeted and switched off, cancer cells that are addicted to this pathway will be disproportionately affected, sparing normal cells (Weinstein, 2002; Weinstein and Joe, 2006, 2008). This is the ideal cancer treatment, with a surgical precision in its action, leaving negligible side effects that biomedical researchers have been working toward for decades.

## Potential Targeting of Atr Isomerization in Cancer Therapies

Prior to the elucidation of this anti-apoptotic role of *cis*-ATR in the cytoplasm, a wealth of knowledge already existed about the nuclear kinase roles of ATR which is a *trans* isomer and several cancer therapies have taken advantage of this by targeting the kinase function of ATR to promote cancer cell killing. ATR inhibitors, in combination with chemo- and radio-therapy, have been utilized in a synthetic lethality approach to sensitize cancer cells for cell death with varied results (Wagner and Kaufmann, 2010; Toledo et al., 2011; Fokas et al., 2014; Karnitz and Zou, 2015; Lecona and Fernandez-Capetillo, 2018). Challenges to this approach include: development of specific ATR inhibitors, delivery of the ATR inhibitors to achieve useful physiological concentrations in test subjects, and specificity in killing only cancer cells and not normal cells. VX-970, AZD6738, and other ATR inhibitors are in ongoing clinical trials, being used in conjunction with chemo- or radio-therapy for breast (Kim et al., 2017), ovarian (Huntoon et al., 2013), pancreatic (Prevo et al., 2012), and small cell lung cancers (Vendetti et al., 2015). Pin1 inhibitors also are being evaluated for their usefulness in cancer therapies (Zannini et al., 2019); however, it is possible that side effects could be a concern for this targeting due to the number and diversity of important Pin1 substrates in the cell.

**FIGURE 5:**
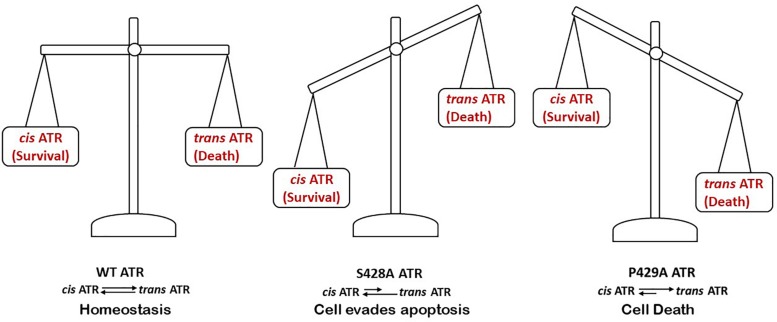
An appropriate balance between cytoplasmic levels of *cis-* and *trans-* ATR is critical for the wellbeing of cells.

It should be pointed out that the current ATR inhibitors used in cancer clinical trials are specific inhibitors of ATR kinase activity which is pivotal to the hallmark ATR’s DNA damage checkpoint functions in the nucleus. Since the new anti-apoptotic activity of *cis*-ATR at mitochondria is independent of ATR kinase activity (Hilton et al., 2015), these inhibitors have no effect on *cis*-ATR’s anti-apoptotic activity. *Cis*-ATR (ATR-H), potentially, can be such a target protein that is novel and could be effective in cancer treatment. *Cis*-ATR is not directly mutagenic, but it allows cancer cells to evade apoptosis, a very important hallmark of carcinogenesis. It is possible that cancerous cells, especially with chemo- or radio-therapeutic challenge, have a proportionally higher level of cytoplasmic *cis-*ATR and are resistant to killing due to a low level of Pin1 or a lower level of the phosphorylation of Ser428 in ATR than normal cells (Ibarra et al., 2017). In support, a reduced level of pSer428 ATR in the cytoplasm of advanced stage epithelial ovarian cancer cells correlates with a poor prognosis (Lee et al., 2015). Therefore, targeting *cis*-ATR as an adjuvant in treating cancers by irradiation or chemotherapy should preferentially kill *cis*-ATR-addicted cancer cells, with minimal effects on the normal functions of nuclear *trans*-ATR in cells. ATR is an essential protein (Brown and Baltimore, 2000) and its *cis* and *trans* isomers function normally and exist in a delicate balance to ensure cellular survival and normality (Figure 5). By utilizing the natural balance that exists in normal human cells between *cis-* and *trans-*ATR isoforms, we propose *cis*-ATR as a novel, potential target in cancer treatment. Also, *cis*-ATR might serve as a diagnostic marker of prognosis and treatment efficacy in cancer management.

Given the critical role of Pin1 in maintaining the balance between *cis*- and *trans*-ATR in the cytoplasm, manipulation of Pin1 subcellular level or activity could be another means to control *cis*-ATR formation for cancer therapeutics. Ibarra et al. recently reported different subcellular distribution of Pin1 in different cell types in zebrafish *in vivo*, suggesting specific mechanisms for regulating Pin1 subcellular activity are cell-type dependent (Ibarra et al., 2017). These authors also found dramatic reduction of Pin1 in the nucleus and high cytoplasmic Pin1 levels in some cell types *in vivo* (Ibarra et al., 2017). These findings could have important implications in terms of cytoplasmic *cis*-ATR formation.

## Prospective

There are still important questions remaining to be answered to validate the hypotheses put forward in this review, including a better understanding of (1) how the Ser428 residue is phosphorylated or dephosphorylated under different physiological and biological conditions. Phosphorylation status plays a critical role in the regulation of ATR isomerization and, thus, its antiapoptotic activities; (2) the structural differences between the *cis* and *trans* isomers; and (3) their specific folding for substrate recognition and binding. Are there specific binding partners of *cis-* and *trans-*ATR in the cytoplasm and nucleus, respectively, which help to energetically stabilize ATR in their isoforms? If so, what are these proteins and how are they regulated. Understanding the mechanisms of each isomer’s formation and stabilization can help to define whether *cis*-ATR fulfils the criteria to be termed an oncoprotein. It also should be possible to develop drugs that can selectively increase or reduce the specific ATR isoform that is needed in the management of a disease, as elucidated earlier for cancer, for example.

The quest for an ideal cancer therapy began when cancer itself was described as a disease and many promising targets have been investigated in the past with varying results. Since a cancer cell starts as a normal cell that has become deregulated, the ability to selectively target only cancer cells by identification of proteins/processes unique to cancer cells remains elusive for many cancer types and stages. Such targeting should minimize adverse effects while obtaining an effective treatment. As a further complication, the pathways that lead to cancer are numerous and varied, with confounders like immunoediting, persistence of cancer stem cells, etc. Here we propose a target common to all cells: isomerization-mediated apoptosis, but in such a specifically targeted way that normal cells are spared. The isomerization of ATR by Pin1 is an important biological process that should be studied further since the existing evidence points to exciting possibilities for drug/genetic regulation of this singular process. There would be significant potential translational implications in disease diagnosis and treatment.

Finally, the ability to induce or prevent apoptosis in select groups of cells can be of importance in other diseases such as ischemia and inflammation where cell death is the major issue. Moreover, it is worth investigating if *cis*-ATR plays a role in elongating the life of a cell in the context of aging since more cells would be able to successfully evade apoptosis by increasing the mitochondrial health of the cell.

## Author Contributions

YM wrote the draft of the manuscript based on the outlines made by YZ. YZ oversaw the process. All authors read and participated in revising the manuscript.

## Conflict of Interest

The authors declare that the research was conducted in the absence of any commercial or financial relationships that could be construed as a potential conflict of interest.
